# Action Potential Energy Efficiency Varies Among Neuron Types in Vertebrates and Invertebrates

**DOI:** 10.1371/journal.pcbi.1000840

**Published:** 2010-07-01

**Authors:** Biswa Sengupta, Martin Stemmler, Simon B. Laughlin, Jeremy E. Niven

**Affiliations:** 1 Neural Circuit Design Group, Department of Zoology, University of Cambridge, Cambridge, United Kingdom; 2 BCCN Munich, LMU München, Martinsried, Germany; 3 Smithsonian Tropical Research Institute, Panamá, República de Panamá; University College London, United Kingdom

## Abstract

The initiation and propagation of action potentials (APs) places high demands on the energetic resources of neural tissue. Each AP forces ATP-driven ion pumps to work harder to restore the ionic concentration gradients, thus consuming more energy. Here, we ask whether the ionic currents underlying the AP can be predicted theoretically from the principle of minimum energy consumption. A long-held supposition that APs are energetically wasteful, based on theoretical analysis of the squid giant axon AP, has recently been overturned by studies that measured the currents contributing to the AP in several mammalian neurons. In the single compartment models studied here, AP energy consumption varies greatly among vertebrate and invertebrate neurons, with several mammalian neuron models using close to the capacitive minimum of energy needed. Strikingly, energy consumption can increase by more than ten-fold simply by changing the overlap of the Na^+^ and K^+^ currents during the AP without changing the APs shape. As a consequence, the height and width of the AP are poor predictors of energy consumption. In the Hodgkin–Huxley model of the squid axon, optimizing the kinetics or number of Na^+^ and K^+^ channels can whittle down the number of ATP molecules needed for each AP by a factor of four. In contrast to the squid AP, the temporal profile of the currents underlying APs of some mammalian neurons are nearly perfectly matched to the optimized properties of ionic conductances so as to minimize the ATP cost.

## Author Summary

Neurons produce a myriad of action potentials with different shapes and varying heights and widths; underlying these action potentials are highly nonlinear, voltage-dependent ionic conductances with varying biophysical properties. Each action potential comes at a cost: the brain uses a substantial portion of its total energy budget to generate and propagate action potentials. Recent results show that some mammalian action potentials have biophysical properties that make them energy efficient. Yet, how widespread are energy efficient action potentials? Using mathematical analysis and modeling, we show that there is no direct relationship between the height, width, and the energy consumption of a single action potential. Furthermore, we establish that many mammalian action potentials have biophysical properties that reduce the overlap between their inward and outward currents so as to minimize energy consumption. This reduction in overlap results from a combination of ion channel properties uniquely tailored for each particular neuron type and the functional purpose of the action potential in that neuron. By comparing the measured biophysical parameters to the parameters produced by numerical optimization for maximal energy-efficiency, we argue that natural selection for energy-efficiency could help explain both the shape of the action potential and the underlying biophysics of ionic currents.

## Introduction

Electrical signaling within neurons dominates the energy consumption of mammalian brains [1], [2] and action potentials (APs) make a significant contribution to overall usage [1]. The energy consumption of APs is due to the influx of Na^+^ ions and efflux of K^+^ ions through voltage-gated ion channels, which charge the membrane capacitance to the peak of the AP and then discharge it back to resting potential. To maintain signaling the Na^+^/K^+^ ATPase pumps these ions back across the membrane using energy provided by ATP [3]. There are three basic reasons why APs use significant quantities of energy. First, to make a robust signal, the membrane capacitance is usually charged by more than 50 mV to the peak of the AP. Second, because APs travel considerable distances along densely packed axons, collaterals and dendrites, the total area of membrane invaded by APs is large and so, therefore, is the capacitance that must be charged to the peak voltage. Third, the flux of Na^+^ and K^+^ ions exceeds the minimum required to charge the membrane to peak potential because the Na^+^ and K^+^ currents overlap [4], [5].

The high energy cost of transmitting APs in the central nervous system is thought to have influenced the structure of neural codes and circuits [6]. Redundancy reduction and sparse codes reduce the number of APs required to represent information and efficient wiring reduces the capacitance of neural circuits by minimizing the distance over which APs must propagate. It is also possible to reduce energy consumption by making APs energy efficient [7], [8]. For example, in hippocampal mossy fiber axons the biophysical properties of the voltage-gated Na^+^ and K^+^ channels generating the AP are adjusted to reduce the flow of Na^+^ and K^+^ ions across the membrane [7]. The activation, inactivation and deactivation of the mossy fiber's channels are particularly rapid, leading to a very brief AP, with a half-width of 250 µs. These rapid channel kinetics reduce the overlap between Na^+^ and K^+^ currents, thereby reducing the number of Na^+^ ions entering the axon during the AP to just 1.3 times the amount required to charge the membrane capacitance [7].

The waveform of the mossy fiber AP is very different from the squid giant axon's, which has an amplitude of 100 mV, a half-width of 1.5 ms and, because of extensive overlap between Na^+^ and K^+^ currents, produces a Na^+^ influx that is close to 4 times the capacitive minimum [5]. A wide variety of AP shapes have been recorded in vertebrate and invertebrate neurons (e.g. [7]–[14]). Carter and Bean [8] have suggested that the primary determinant of differences in Na^+^ entry efficiency among neurons is their different AP shapes rather than Na^+^ channel kinetics. This raises a number of questions. How energy efficient are APs in vertebrate and invertebrate neurons? Is the reduction in the overlap of the Na^+^ and K^+^ currents a general mechanism for improving the energy efficiency and are other means, such as reducing AP width and amplitude, also effective? What properties of voltage-gated ion channels are necessary to produce energy efficient APs? The answers to these questions have important consequences for bottom-up energy budgets of the mammalian brain as well as estimates of the numbers of APs that they can support [1]. These in turn affect the interpretation of non-invasive brain imaging methods that infer neural activity from local changes in energy consumption.

We use single compartment models to assess the energy consumption of APs from seven neurons from both vertebrates and invertebrates. The energy costs of these APs vary by over an order of magnitude from the most economical, the rat granule cell and mouse thalamo-cortical interneuron, to the most profligate, the squid giant axon. We developed optimization methods based on perturbation theory to alter the number and/or biophysical properties of the Na^+^ and K^+^ voltage-gated ion channels, including their conductance per unit area and time constants, to find the minimum energy consumption of APs in six of these neuron models. In thalamo-cortical interneurons, hippocampal interneurons and cerebellar granule cells the energy consumption of APs measured experimentally is close to the minimum predicted by our constrained optimization algorithm, and to the minimum Na^+^ entry needed to charge the membrane capacitance. Conversely, the energy consumption of APs in a crab motor neuron and the squid giant axon is far from the minimum achievable, and further still from the capacitive minimum. Comparison among our optimized models shows that the most energy efficient APs are generated by Na^+^ and K^+^ currents that have a substantially reduced overlap than those recorded experimentally. We argue that functional constraints prevent many neurons from using the most energy efficient APs but that in some situations APs approach their optimum energy efficiency.

## Results

### Energy cost of Action Potentials (AP) in single compartment models

**Figure 1:**
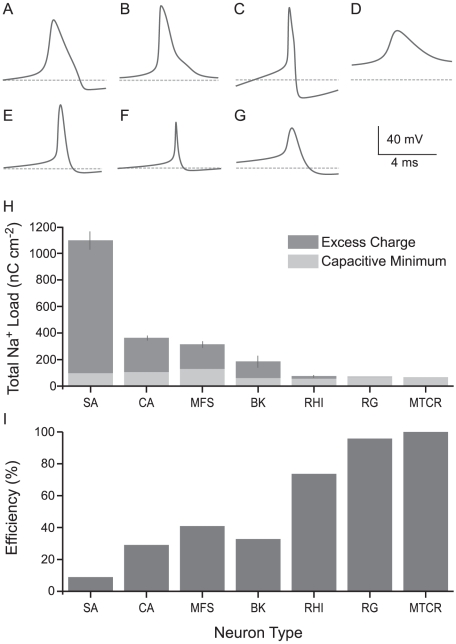
Action potential energy usage in seven neuron models from vertebrates and invertebrates. (A) The shapes of action potentials in single compartment Hodgkin-Huxley type models from the squid giant axon (SA), (B) crab motor neuron axon (CA), (C) mouse fast-spiking neuron (MFS), (D) honeybee Kenyon cell (BK), (E) rat hippocampal interneuron (RHI), (F) rat granule cell (RG) and (G) mouse thalamo-cortical relay neuron (MTCR). The dashed grey line indicates the resting potential of each model. (H) The respective Na^+^ load (nC cm^−2^) of each action potential (dark grey) and the capacitive minimum Na^+^ load of each action potential (light grey). Error bars show the effect of changing the peak conductances of the voltage-gated ion channels by ±5% on AP energy consumption. (I) The efficiency of action potentials from each model. Error bars show the effect of changing the peak conductances of the voltage-gated ion channels by ±5% on AP energy efficiency.

Neurons contain a variety of voltage-gated ion channels that contribute to action potential (AP) generation [15]. In several neurons the properties of these ion channels, including their density, single channel conductance, activation/inactivation kinetics and voltage dependency, have been measured and then incorporated into models to demonstrate that each neuron is using its combination of channels to produce its particular AP. To compare the energy costs of different APs we constructed published single compartment models of seven of these neurons, including examples from invertebrates and vertebrates (Figure 1). The models were of the squid (*Loligo fobesi*) giant axon, SA [12], a crab (*Cancer magister*) leg motor neuron axon, CA [9], a mouse (*Mus musculus*) fast spiking GABAergic cortical interneuron, MFS [10], a worker honeybee (*Apis mellifera*) mushroom body Kenyon cell, BK [16], a rat (*Rattus norvegicus*) hippocampal interneuron, RHI [14], a rat cerebellar granule cell, RG [13], and a mouse thalamocortical relay neuron, MTCR [11] (Figure 1).

The different combinations of conductances (Table S1) in these neurons produce AP's with different waveforms and excitabilities (Figure 1A). Every model has three basic conductances, a leak conductance, a voltage-gated Na^+^ conductance and a voltage-gated K^+^ conductance, all of which have properties particular to each model (Table S1). Two models, the squid giant axon model [12] and the hippocampal GABAergic interneuron model [14] use just these three conductances. The other models contain more conductances that produce additional currents. Three models have one additional current; the crab motor neuron an A-type K^+^ current [9]; the fast spiking cortical interneuron a slowly inactivating D-type K^+^ current [10], and the thalamocortical relay neuron model a low-threshold Ca^2+^ current [11]. The honeybee Kenyon cell model has three additional currents, a second voltage-gated Na^+^ current, as well as an A-type and a slow transient K^+^ current [16]. The cerebellar granule cell model includes four additional currents, an A-type, a C-type and a hyperpolarization-activated K^+^ current, and an L-type Ca^2+^ current [13] (Table S1). Even in cerebellar granule cell or thalamocortical relay neuron models, which possess inward currents in addition to the inward Na^+^ current, 95 and 92% respectively of the energy consumed by the AP is used to extrude Na^+^ ions entering through voltage-gated Na^+^ channels. Additional inward currents, such as T-type Ca^2+^ channels in thalamocortical relay neuron model, consume energy because each Ca^2+^ ion is exchanged for three Na^+^ ions by the Na^+^/Ca^2+^ exchanger [17], [18] and these Na^+^ ions must then be extruded by the Na^+^/K^+^ ATPase.

We derived the energy consumption of a single AP from the Na^+^ influx, which was determined by integrating the Na^+^ current over a single period of repetitive firing (Figure 1B). Because these are deterministic models the contributions of the sub-threshold Na^+^ currents that flow between APs are negligible [4]. The Na^+^/K^+^ ATPase extrudes the Na^+^ ions entering the neuron during the AP to maintain the Na^+^ concentration gradient across the membrane using the energy liberated by the hydrolysis of ATP to ADP [1], [3].

The combination of conductances in each model determines the total Na^+^ influx and, hence, its ATP consumption. Consumption differed widely among our 7 models (Figure 1B, Table 1). At one extreme the Na^+^ charge transfer per unit membrane area (hereafter the Na^+^ load) of an AP in the squid giant axon at 6.3°C was 1098 nC cm^−2^, consuming 2.3*10^12^ ATP molecules cm^−2^. At the other extreme the Na^+^ load of an AP in the mouse thalamocortical relay neuron was 17 times less, just 65 nC cm^−2^ consuming 1.35*10^11^ ATP molecules cm^−2^. Three other models of both vertebrate (rat hippocampal interneuron and rat granule cell) and invertebrate neurons (bee Kenyon cell) have low energy consumption APs, with a Na^+^ load of below 200 nC cm^−2^, whereas the mouse fast spiking interneuron and crab motor neuron APs have higher Na^+^ loads of 315 and 364 nC cm^−2^, respectively (Figure 1B; Table 1). These calculations show that AP energy consumption differs widely among our models, with low energy consumption APs being found in both vertebrate and invertebrate neurons. Six of our models fall within a six-fold range of AP energy consumption, with the squid giant axon AP as an outlier (Figure 1B, Table 1).

**Table 1:** Properties of action potential waveform of single action potentials from the seven single compartment models.

	SA	CA	MFS	BK	RHI	RG	MTCR
**Original Na^+^ load [nC cm^−2^]**	1098	364	315	186	76	72	65
**Original AP height [mV]**	98	106	129	61	55.5	69	65
**Original AP full-width at half maximum [ms]**	1.47	0.93	0.47	2.6	0.65	0.2	1.1
**Original overlap [nC cm^−2^]**	1034	264	231	129	36	9	15
**Optimum Na^+^ load [nC cm^−2^]**	263	169	225	121	59	-	65
**Optimum overlap [nC cm^−2^]**	185	31	133	42	23	-	15
**Capacitive minimum [nC cm^−2^]**	98	106	129	61	56	69	65

The biophysical parameters for our single compartment models were taken from previous publications [9]–[14], [16]. Only two of these studies [12], [16] specified the errors that were associated with the experimental determination of these parameters. We incorporated a ±5% error into the peak conductances of our models to determine their effect on the Na^+^ load of the APs and, hence, their energy consumption (Table S2). Introducing these errors into the models made only a small difference (typically <6%) to the Na^+^ load of each of the APs (Figure 1B), suggesting that AP energy consumption is truly different for each of the models.

### The origins of differences in energy cost

**Figure 2:**
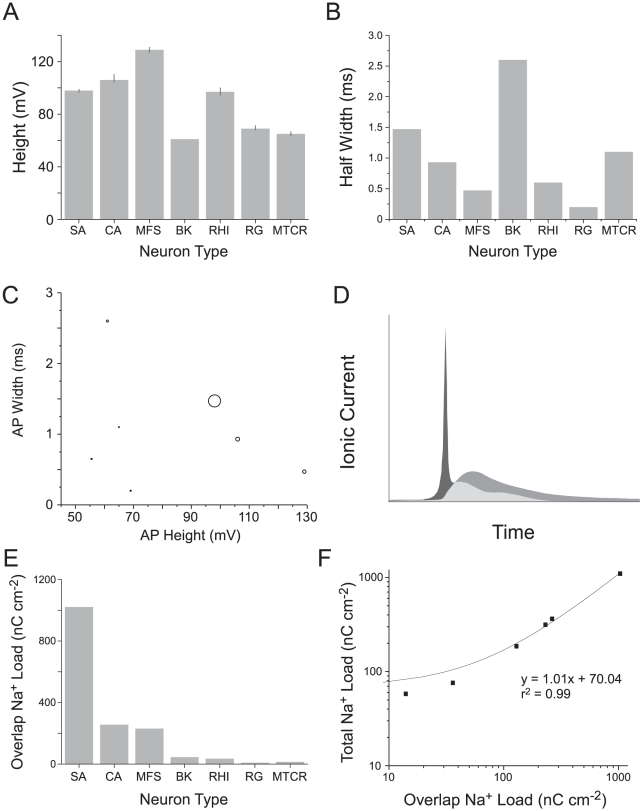
The overlap between Na^+^ and K^+^ currents affects action potential efficiency. (A) The heights of action potentials in the seven models. Error bars show the effect on AP height of changing the peak conductances of the voltage-gated ion channels by ±5%. (B) The half widths of action potentials in the seven models. Error bars are not visible because changing the peak conductances of the voltage-gated ion channels by ±5% did not affect AP width. (C) The relationship between the height, width and Na^+^ load of action potentials from each of the models shown in Figure 1. The size of each circle is proportional to the energy consumed by the action potential. (D) Schematic diagram of the ionic charge distributions of an action potential depolarizing charge (horizontal lines), overlap charge (hatched) and hyperpolarizing charge (vertical lines). (E) The overlap charge in action potentials from each of the seven models. (F) The relationship between the overlap charge and the total Na^+^ load. SA, squid giant axon; CA, crab motor neuron; MFS, mouse fast spiking neuron; BK, honeybee Kenyon cell; RHI, rat hippocampal interneuron; RG, rat granule cell; MTCR, mouse thalamocortical relay neuron.

The primary purpose of the nonlinear, AP-generating Na^+^ current is to charge the membrane to the peak of the AP. To load the capacitive charge onto the membrane, the local Na^+^ current and the current of synaptic origin flowing along the dendrite-to-axon axis both contribute; the latter current is represented by the constant injection current in the models. During the charging of the membrane, the sum of currents must also overcome any resistive losses through the membrane. For a given AP waveform and a given set of passive neuronal parameters (specific membrane capacitance and resistance), one can compute the active Na^+^ current that will minimize Na^+^ accumulation inside the cell. As a rule of thumb, for a cell with high input resistance and low AP threshold, the capacitive charge and the minimal Na^+^ load are nearly equal. Hence, the minimum Na^+^ load scales with the AP height. Assuming that specific membrane capacitance is constant, this minimum Na^+^ load varies by a factor of approximately 2.3, because the model APs range in height from 55.5 mV to 129 mV (Figure 2A).

The other factor in the AP energy cost is efficiency. Efficiency is the ratio (in percent) between the capacitive minimum Na^+^ load and the total Na^+^ load (Figure 1B,C). Hodgkin (1976) observed that in squid giant axon at 18°C the temporal overlap between inward Na^+^ current and outward K^+^ current greatly reduced efficiency to around 25%, although a recent modification of the squid model suggests an efficiency of 40% [4]. By comparison some mammalian neurons approach the theoretical minimum Na^+^ load with efficiencies approaching 100% [7], [8]. Our modeling of the mouse thalamocortical relay neuron (MCTR) and rat granule cell (RG) demonstrates that different combinations of conductances can be used to achieve efficiencies close to 100%, and the rat hippocampal interneuron (RHI) achieves an efficiency of 75%. The efficiencies of the other APs are substantially lower; 3 models fall in the range of 20%–40% and at 9%, the squid giant axon at 6.3°C (SA) is particularly inefficient (Figure 1C). Note that among our 7 models the 3 most energy efficient APs also have low theoretical minimum Na^+^ loads because they have low heights (Figure 1A,C)

### Temperature and efficiency

Changing the temperature at which the simulations are executed influences both the Na^+^ load of the AP and, by changing the amplitude, the capacitive load. At 18°C the squid giant axon Na^+^ load is 331 nC cm^−2^, less than one third of the 1098 nC cm^−2^ that we calculate at 6.3°C. The Na^+^ load is 3.85 times the capacitive load of 86 nC cm^−2^, close to value calculated by Hodgkin [5] at the same high temperature but higher than the value calculated by Crotty *et al.* [4] using a modified Hodgkin-Huxley model. At 6.3°C the capacitive load is higher because the AP is taller, and the total Na^+^ load is 11.2 times this load. Thus lowering the temperature slightly increases the capacitive load but massively reduces efficiency. We show below that efficiency is lowered by increasing the time constants of voltage-gated conductances.

### Energy efficiency and AP shape

Both the energy consumption and the shape (height and width) of an AP are determined by the currents that generate the AP, and a comparison of 4 types of mouse CNS neurons has recently shown that energy consumption increases as AP width decreases [8]. Is there a clear relationship between width, height and energy consumption among our 7 models? The models encompass a 2.3-fold range of heights, from 55.5–129 mV, and a 13-fold range of durations, from 0.2 ms to 2.6 ms (Figure 2A,B; Table 1). Over this range there is a tendency for energy consumption to increase with height, but there is no obvious relationship between width and energy consumption (Figure 2C). We also incorporated a ±5% error into the peak conductances of our models to determine their effect on AP height and half-width. Introducing errors into the models a small difference to the height (<5%) but not to the width of each of the APs (Figure 2A,B). Thus, AP height and width are robust to small changes in the underlying conductances.

### Overlap load dominates energy efficiency

Our models confirm that the overlap between the Na^+^ and K^+^ currents that generate the AP (Figure 2D) is the major determinant of efficiency. Overlap makes the squid AP inefficient [4], [5] and efficient AP's have little overlap [7], [8] (Figure 2E). Following Crotty *et al.* [4], we calculated the overlap load as the difference between the total Na^+^ current and the depolarizing component of the Na^+^ current (Figure 2D, also see Figure 3 in [4]). Among our models there was a greater that 100-fold difference in the overlap load from 9 nC cm^−2^ in the rat granule cell model to 1034 nC cm^−2^ in the squid giant axon model at 6.3°C (Figure 2E), and the overlap load is linearly related to the total Na^+^ load with a slope close to 1 (R^2^ = 0.99; p<0.0001) (Figure 2F).

**Figure 3:**
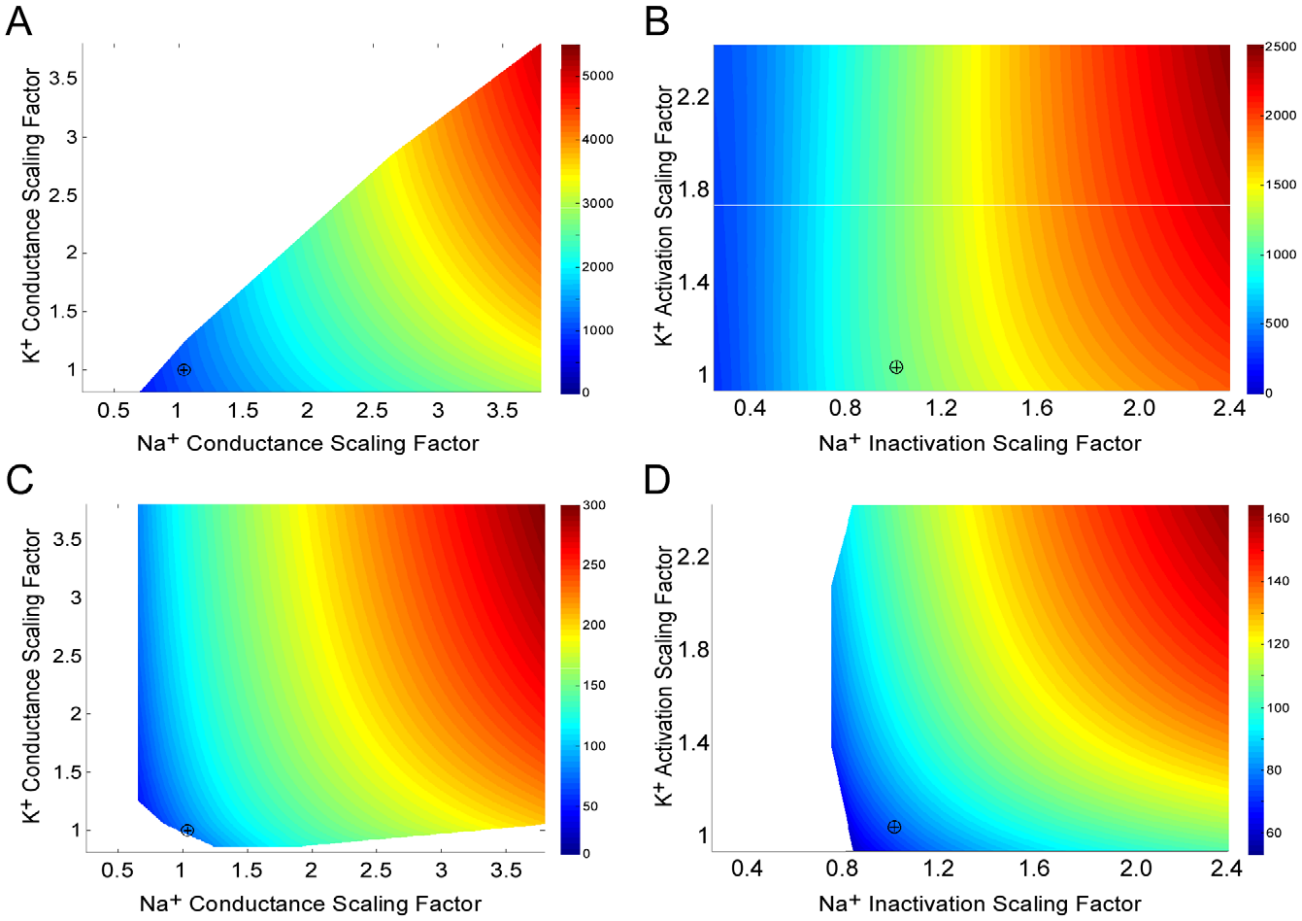
Scaling biophysical properties of voltage-gated ion channels influences action potential energy consumption. (A) Scaling the conductance per unit area of Na^+^ (g_Na_) and K^+^ (g_K_) channels affects the Na^+^ load of the action potential of the squid giant axon (SA) model. (B) Scaling the inactivation (τ_h_) time constant of the Na^+^ voltage-gated channel and the activation (τ_n_) time constant of the K^+^ voltage-gated channel affects the Na^+^ load of the action potential of the squid giant axon (SA) model. (C) Scaling the conductance per unit area of Na^+^ (g_Na_) and K^+^ (g_K_) channels affects the Na^+^ load of the action potential of the rat hippocampal interneuron (RHI) model. (D) Scaling the inactivation (τ_h_) time constant of the Na^+^ voltage-gated channel and the activation (τ_n_) time constant of the K^+^ voltage-gated channel affects the Na^+^ load of the action potential of the rat hippocampal interneuron (RHI) model. Colors indicate the Na^+^ load of a single AP (nC cm^−2^). ‘+’ indicates the Na^+^ load of the original model.

### Biophysical basis of AP energy efficiency

We investigated how the properties of voltage-gated conductances influence efficiency by, for example, determining the overlap load. We systematically varied either conductance per unit area or activation/inactivation time constants in the squid giant axon model (Figure 3). The measured values that defined the original model were increased or decreased by multiplying them with a scaling factor (see Methods). Because it is assumed that the single channel conductance remained constant, varying the conductance per unit area is equivalent to changing the channel density. Changes in channel density affect the gating charge, which could influence the total Na^+^ load of the APs; however, our modeling shows that the effect of gating charge on AP Na^+^ load is small (see below). This agrees with the observation made by Crotty *et al.* [4] that the gating current changes the ionic fluxes by less than 5%. Reducing the Na^+^ and K^+^ conductances per unit area revealed a small region of parameter space in which APs had a lower Na^+^ load than the original (Figure 3A) because the voltage-gated currents are reduced. Conversely, increasing the Na^+^ or K^+^ conductance per unit area produced APs with a higher Na^+^ load (Figure 3A). Parameter combinations in which the K^+^ conductance per unit area was increased more than that of the Na^+^ conductance per unit area generally prevented the model from generating APs (blank zone in Figure 3A).

We then scaled the inactivation time constant (τ_h_) of the voltage-gated Na^+^ conductance against the activation time constant (τ_n_) of the voltage-gated K^+^ conductance in the squid giant axon model. This scaling revealed a large region of parameter space with shorter Na^+^ inactivation time constants (τ_h_), where the Na^+^ load was lower than the original (Figure 3B) because the Na^+^ current terminated more rapidly. Conversely, the Na^+^ load was increased at longer τ_h_ (Figure 3B). Longer K^+^ activation time constants increased AP Na^+^ load when τ_h_ was also longer, but had very little effect with shorter τ_h_ (Figure 3B). When the K^+^ activation time constants were reduced to below the plotted values (Figure 3B) the model demonstrated mixed-mode oscillations and period-doubling towards chaos [19]. In contrast to scaling conductance per unit area, scaling the channel time constants did not reveal large areas of parameter space in which the membrane was inexcitable. In conclusion, lowering conductances per unit area and shortening channel time constants both produce squid APs with lower Na^+^ loads, and hence energy consumption, but changing the time constant is much more effective (Figure 3A,B).

We also scaled the conductance per unit area (Figure 3C) and the activation/inactivation time constants of the Na^+^ and K^+^ conductances (Figure 3D) in the rat hippocampal interneuron model. Again, decreasing the Na^+^ or K^+^ conductance per unit area produced APs with a lower Na^+^ load and, therefore, a lower energy cost than the original (Figure 3C). So, too, did shortening the Na^+^ inactivation time constant, although this region of parameter space was much smaller than in the squid giant axon model (Figure 3D). Within the parameter space we explored, scaling of the conductance per unit area or channel time constants in the hippocampal interneuron model produced regions that failed to support APs (Figure 3D). These observations suggest that the higher efficiency of the rat hippocampal interneuron is achieved by reducing the Na^+^ inactivation time constant to a value that approaches its lower limit for excitability.

### Relationships between AP waveform and efficiency within models

**Figure 4:**
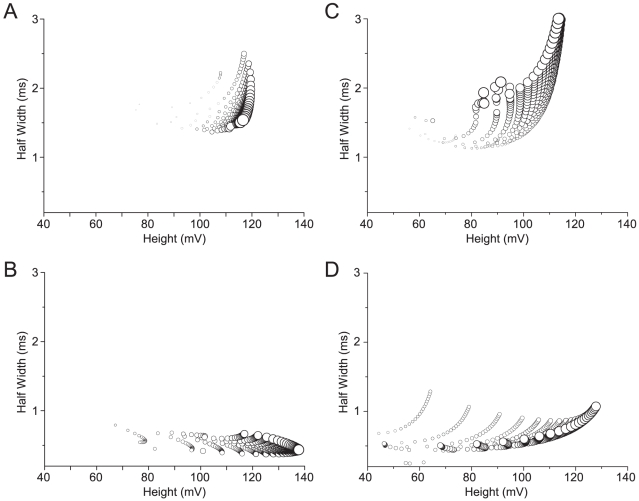
The relationship between action potential height, width and energy consumption within a neuron model. (A) The relationship between action potential height, width and Na^+^ load when the conductances of the Na^+^ and K^+^ voltage-gated channels are scaled in the squid giant axon (SA) model. In all the panels the size of the circle is proportional to the Na^+^ load of a single AP, larger circles indicating higher action potential Na^+^ load. (B) The relationship between action potential height, width and Na^+^ load when the time constants (τ_h_, τ_n_) of the Na^+^ and K^+^ voltage-gated channels are scaled in the squid giant axon (SA) model. (C) The relationship between action potential height, width and Na^+^ load when the conductances of the Na^+^ and K^+^ voltage-gated channels are scaled in the rat hippocampal interneuron (RHI) model. (D) The relationship between action potential height, width and Na^+^ load when the time constants (τ_h_, τ_n_) of the Na^+^ and K^+^ voltage-gated channels are scaled in the rat hippocampal interneuron (RHI) model.

Changing the conductance per unit area or the activation/inactivation time constants of the voltage-gated ion channels usually changes the shape of the AP (e.g. [4], [20]). Consequently the results from our parameter variations allowed us to examine the relationship between AP height, width and Na^+^ load within a neuron model (Figure 4). In the squid giant axon, changing the conductance per unit area or activation/inactivation kinetics of the voltage-gated ion channels affected AP height, width and Na^+^ load (Figure 4 A,B). At a particular height, the most expensive APs are the widest, while taller APs tend to be more expensive than their shorter counterparts (Figure 4A,B). In the rat hippocampal interneuron, the main consequence of scaling the conductance per unit area or the activation/inactivation kinetics was, like squid, to produce taller APs with higher energy consumption (Figure 4C,D). However, when time constants were varied the wider APs almost invariably consumed less energy (Figure 4D); the opposite of what we observed in the squid giant axon (Figure 4B). This comparison demonstrates that relationships between height, width and energy consumption are strongly model dependent.

This model dependency is hardly surprising because many different combinations of outward and inward currents can produce the same net current, and hence rate of change of membrane potential. To prove this point we adjusted the conductance per unit area and the activation/inactivation kinetics of the voltage-gated ion channels to produce APs with identical height and width but very different currents and hence energy consumptions (Figure S1). Thus shape alone is not generally a reliable indicator of an AP's energy cost.

### Constrained optimization of AP energy efficiency

In the previous section we varied pairs of parameters while holding all others constant but this is an unnatural restriction. A more searching analysis, in which we test all possible combinations of parameters (e.g. [20]) to find the most energy efficient AP, was not feasible because it would involve more parameter combinations than we could reasonably compute. Instead, we used numerical methods for constrained optimization to traverse through the high-dimensional parameter space. This procedure enabled us to simultaneously vary several parameters and find values that reduce the AP Na^+^ load heuristically, without imposing excessive computational demands. The heuristic search combines two standard approaches to finding an optimum in a bounded parameter space. First a gradient-free simplex approach searches the space, and quickly finds a set of parameter values that minimizes energy consumption. Then this “simplex optimum” is used as the starting point for a gradient descent method that searches the bounded parameter space exhaustively, to find the global optimum. The advantage of starting at the “simplex optimum” is that it greatly reduces the time taken to complete the exhaustive search. The search procedures were carefully designed to take account of two factors (see Methods). First, the search algorithm can enter regions of parameter space for which the input produces no APs and hence no measure of AP energy consumption is available. This is because the membrane is inexcitable. Second, the AP height had to be kept within narrow bounds to prevent optimization driving the AP amplitude down, eventually to zero.

Using this optimization method we minimized AP Na^+^ load by simultaneously varying five parameters, the conductances of the Na^+^ and K^+^ voltage-gated ion channels, the Na^+^ channel activation and inactivation time constants (τ_m_, τ_h_) and the K^+^ channel activation time constant (τ_n_). In each of the models we optimized, the conductances of the Na^+^ and K^+^ voltage-gated ion channels were constrained to remain within 30% to 400% of the original, published values for each model. Similarly, the activation and inactivation time constants of the Na^+^ and K^+^ voltage-gated ion channels were not allowed to be rescaled to less than 30% or more than 250% of the original values.

First, we ran the squid giant axon model with 20 µA/cm^2^ constant current injection and constrained our optimization to generate the same height as the experimentally measured AP. If the AP height is constrained from below, optimization always matches the AP height to the lower bound imposed. When the constraint on AP height was set to a value lower than the original, the optimized AP was always matched to the height constraint and consumed less energy than APs optimized with higher height constraints. Thus, when the bound on AP height is lowered to 40 mV, our constrained optimization produces an AP of approximately 40 mV, which consumes substantially less energy than APs constrained to 50, 60 or 98 mV (Figure S2), emphasizing the relationship between AP height and minimal energy consumption. The optimized model was derived by driving trains of APs with constant current. We checked that when this optimized model was stimulated by single current pulses that elicited single APs, the same marked decrease in energy cost was observed (data not shown).

**Figure 5:**
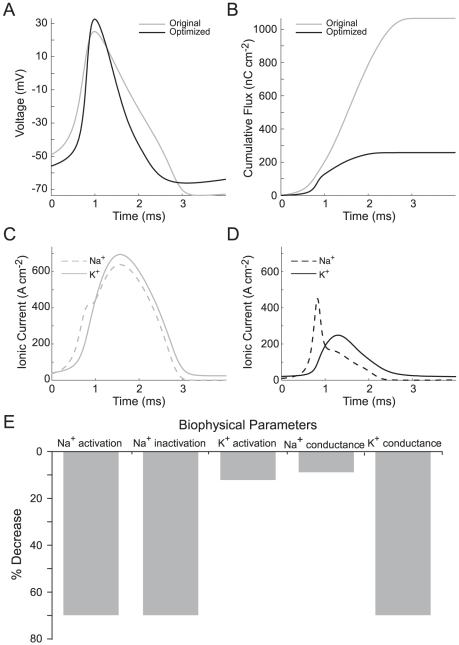
Constrained optimization of the Na^+^ load of the squid axon model produces energy efficient action potentials. (A) The action potential waveform before (light grey) and after (black) optimization. (B) The cumulative Na^+^ load during the action potential before (light grey) and after (black) optimization. (C) The Na^+^ (light grey, dashed line) and K^+^ (light grey, solid line) currents generating the action potential in the original squid giant axon model. Note the extensive overlap between the Na^+^ and K^+^ currents. (D) The Na^+^ (black, dashed line) and K^+^ (black, solid line) currents generating the action potential in the optimized squid giant axon model. Note the reduced overlap between the Na^+^ and K^+^ currents. (E) The changes in each of the biophysical parameters included in our constrained optimization after optimization relative to the original parameter values.

After constrained optimization, the Na^+^ load of the squid giant axon AP at 6.3°C was 263 nC cm^−2^, 78% lower than that of the original (Figure 5A,B). Thus the energy efficiency improved from 9% in the original AP to 37% in the optimized AP. Comparison of the Na^+^ and K^+^ currents that generate the AP before and after optimization showed that the optimized AP had smaller, faster currents with a reduced overlap (Figure 5C,D). The changes in the currents were primarily through a decrease in the Na^+^ channel activation/inactivation time constants and a decrease in the magnitude of the K^+^ conductance (Figure 5E; Table S3). The faster activation/inactivation of the Na^+^ channel enhanced an initial transient increase of the Na^+^ current and reduced the overlap between the Na^+^ and K^+^ currents (Figure 5D). The reduction in the overlap of the Na^+^ and K^+^ currents reduced the Na^+^ flux during the AP, thereby saving energy (Figure 5B).

We also controlled for the fact that gating charge influences the shape of the AP and could affect the minimum energy consumption of the AP after optimization. Gating charge movement causes a transient change in capacitance that precedes the activation, inactivation and deactivation of the channel. This current becomes prominent during the rising foot of the AP [4]. The gating current can be described by a voltage-dependent capacitive current, which we added to our model of the squid giant axon [4]. Repeating the optimization, the parameter set that yields the energy efficient AP is only slightly affected by the inclusion of the gating charge, and the shape of the optimum AP is unaffected (Figure S3).

### Optimized parameters yield energy efficient APs in other neurons

**Figure 6:**
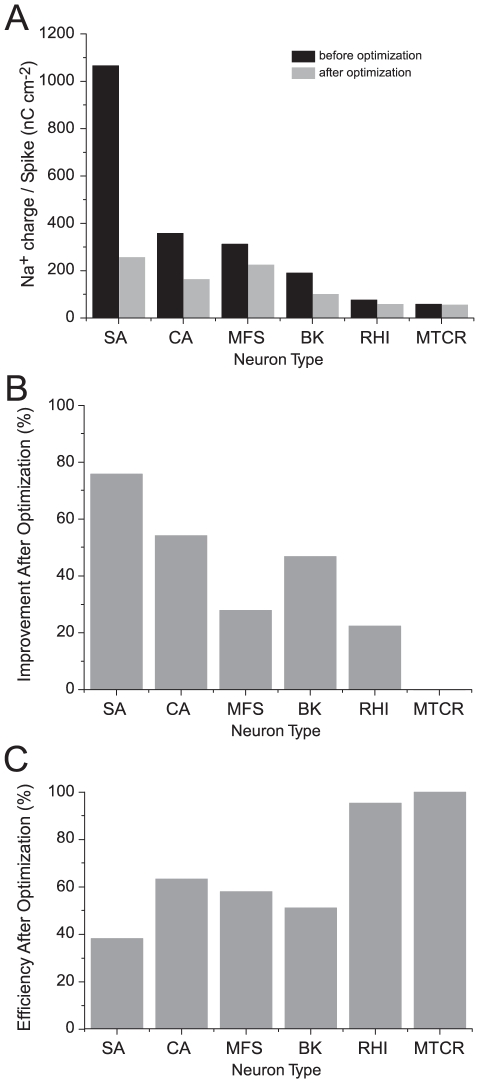
Constrained optimization reduces the energy consumption of action potentials in models of vertebrate and invertebrate neuron models. (A) The Na^+^ load of action potentials in each of the six models before (black) and after (light grey) constrained optimization. (B) The same data as in A, replotted as the percentage improvement in the Na^+^ load of action potentials. (C) The energy efficiency of action potentials in each of the models after optimization. SA, squid giant axon; CA, crab motor neuron; MFS, mouse fast spiking neuron; BK, honeybee Kenyon cell; RHI, rat hippocampal interneuron; RG, rat granule cell; MTCR, mouse thalamocortical relay neuron.

We then optimized five other neuron models (crab motor neuron, mouse fast spiking interneuron, honeybee Kenyon cell, rat hippocampal interneuron and mouse thalamocortical relay neuron) to determine whether biophysical parameters can be found that reduce the Na^+^ loads of these APs. AP height was fixed at the original value in each model. Optimization reduced the Na^+^ load of APs from all of the models by an amount that was model dependent; the largest reductions being made in those models with the highest original Na^+^ load and lowest energy efficiency (Figure 6A,B). After optimization, the energy efficiency of APs from the crab motor neuron, mouse fast spiking interneuron, honeybee Kenyon cell and rat hippocampal interneuron improved to 63% (from 29), 57% (from 41), 50% (from 33) and 95% (from 74), respectively. As predicted, there was hardly any change in the efficiency of the mouse thalamocortical relay neuron, which was already highly efficient (Figure 6C). We also found that the constrained optimization of 5 parameters produced higher energy efficiencies than our attempts to improve efficiency by varying pairs of parameters, confirming the value of the more biologically realistic multivariate approach.

Four of the models we optimized contained conductances in addition to the Na^+^ and delayed rectifier K^+^ conductances. Incorporating these conductances into our optimization might produce APs with lower energy consumption and increase AP energy efficiency. We tested for this possibility in the crab motor neuron model, which contains an additional A-type K^+^ conductance, because this is known to affect AP shape in pyramidal neurons [21] and improve the energy efficiency of photoreceptors in *Drosophila melanogaster* [22]. Nevertheless, constrained optimization incorporating the Na^+^, delayed rectifier and A-type K^+^ conductances produced APs with a Na^+^ load of 150.6 nC cm^−2^. This load is 11% lower than in the AP obtained after optimizing the model with the A-current removed (Figure S4). Thus, additional conductances can contribute to a reduction in AP energy consumption, though in the crab motor neuron the contribution of the A-type K+ conductance is small. This does not exclude the possibility that additional conductances in other neurons may make substantial contributions to reducing the energy consumption and improving the energy efficiency of APs.

**Figure 7:**
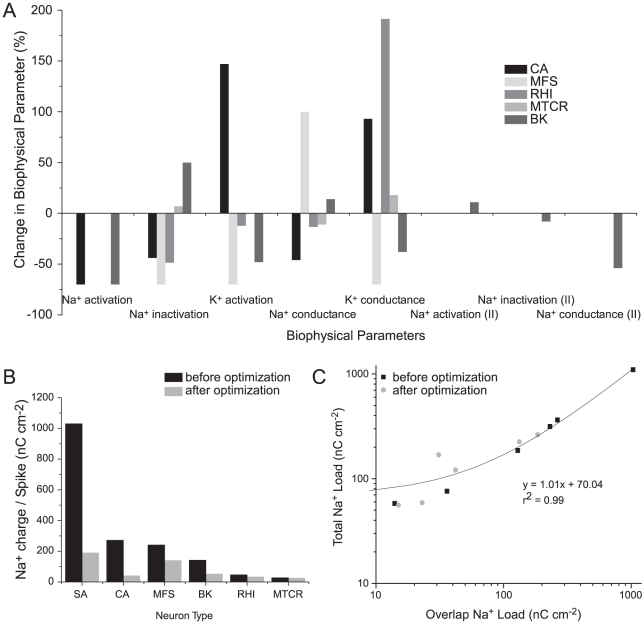
Optimization produces changes in the biophysical properties of voltage-gated ion channels reducing action potential overlap load. (A) Proportional changes in the biophysical parameters of voltage-gated ion channels in five of the models after optimization. Positive values indicate an increase and negative values indicate a decrease in the parameter relative to the original model. (B) The overlap load of each action potential before (black) and after (grey) optimization. (C) The relationship between the overlap load and the total Na^+^ load of action potentials before (black) and after (grey) optimization. SA, squid giant axon; CA, crab motor neuron; MFS, mouse fast spiking neuron; BK, honeybee Kenyon cell; RHI, rat hippocampal interneuron; RG, rat granule cell; MTCR, mouse thalamocortical relay neuron.

We compared models to see whether our constrained optimizations produced similar changes in parameters. All parameters were altered in every model (Figure 7A; Table S3), but there were no obvious common patterns; the same parameter increases in one model and decreases in another. For example, the Na^+^ channel inactivation time constant is shorter in the optimized squid giant axon, crab motor neuron, mouse fast-spiking interneuron and rat hippocampal interneuron models but longer in the optimized bee Kenyon cell model (Figure 5E, 7A). In the bee Kenyon cell, which contain two Na^+^ conductances, we also observed opposite changes in the density and kinetics of these conductances after optimization (Figure 7A). The relative magnitudes of changes vary considerably between models (Table S3). Large changes in both the density and kinetics of Na^+^ and K^+^ channels occur, even in models that already have high efficiency APs before optimization, such as the hippocampal interneuron (Figure 7A), However, in the most efficient neuron, the mouse thalamocortical neuron, the changes in channel densities and kinetics are small.

The common characteristic of optimization, shared by all 5 models, is that the changes in conductances produce faster currents that reduce the overlap load (Figure 7B; Figure S5). Looking across optimized models, the overlap load is positively correlated with the total Na^+^ load of the AP (R^2^ = 0.99; p<0.0001) (Figure 7C). This confirms that the overlap load is the main factor influencing efficiency.

## Discussion

We used seven well-known single compartment models of action potentials (APs) from vertebrate and invertebrate nervous systems (Figure 1, Table 1) to investigate the relationships between the shapes of APs, the currents that generate them and the energy they consume. Energy cost was obtained by integrating the inward Na^+^ current to give the total Na^+^ load, and then calculating the number of ATP molecules that were hydrolyzed by the Na^+^ pump when it ejected this load, operating with a ratio of 3 Na^+^ ions per ATP [1], [4]. These calculations allowed us to compare energy consumption and efficiency among models, identify contributory factors and, by systematically varying parameters within models, determine their effects.

We used single compartment models driven by current to reduce the number of parameters to workable proportions. This restriction limits our findings to the production of brief voltage pulses triggered by injecting current into isopotential cells, but APs rarely occur in such uniform isolation. APs transmit and process information by interacting with other conductances, to integrate inputs and distribute outputs, and often do so in extensive axons and arbors that involve many electrical compartments. However, these interactions are often complicated and particular to specific neurons. We chose to start with a core process in a single compartment to gain a better understanding of basic principles, before working up to more extensive models. In defense of this bottom-up approach, none of our findings contradict previous modeling and experimental studies of the energy efficiency of APs in multi-compartment axons [4], [7].

Our results support previous theoretical work on AP efficiency and energy consumption that used the Hodgkin Huxley (SA) model for propagation along squid giant axon [5], and a modification thereof, the HHSFL (Hodgkin Huxley Sangrey–Friesen–Levy) model (Table S4) [4], [23]. As pointed out by Hodgkin [5] the squid AP is very inefficient, in the sense that the total Na^+^ ion influx (the total Na^+^ load) exceeds the minimum influx that is required to charge the membrane capacitance to the peak of the AP (the capacitive load) (Figure 1). Efficiency is low because the voltage-gated currents of Na^+^ and K^+^ ions overlap (Figure 2,3), allowing the outward K^+^ current to neutralize the charge carried inwards by Na^+^. Efficiency can be increased (Figures 3,5) by decreasing the overlap current and this can be achieved by reducing total conductance via channel density [4] and by reducing the time constants of voltage-gated conductances to shorten the duration of overlap [23]. Consequently when time constants are decreased by raising the temperature at which the model is run, overlap decreases and efficiency improves from 9% at 6.3°C to 24% at 18°C. The HHSFL model returns a higher efficiency, 40%, because its time constants are slightly shorter than the SA, and the delay on the activation of the voltage gated K^+^ conductance is increased [4]. Reducing the time constant of Na^+^ inactivation at constant temperature (Figure 3B,5) is very effective at increasing efficiency [23].

As well as increasing the conduction velocity of axons, higher temperatures reduce noise in axons by reducing channel open times, and hence the rate of spontaneous APs [24]. Our modeling shows that the increased speed of ion channel kinetics at higher temperatures also produces more efficient APs, by reducing overlap between currents at higher temperatures. This combination of reduced noise, increased conduction velocity and improved energy efficiency favors exothermic animals inhabiting warmer environments and might even promote the evolution of endothermy.

We have also extended the analysis of energy efficiency with the squid model by including more parameters, the activation time constants for the voltage-gated Na^+^ and K^+^ conductances, and by simultaneously varying all five of the core parameters, namely the total conductance, activation time constant and inactivation time constant of the voltage-gated Na^+^ conductance, and the total conductance and activation time constant of the voltage-gated K^+^ conductance (Figure 5). Some parameter combinations may not be achieved by ion channels (e.g. large conductance, fast inactivation) due not only to functional constraints, such as producing an AP or maintaining the resting potential [25], but also to molecular constraints on channel proteins. Although we imposed functional constraints on the biophysical parameters of the models during the directed search, they had to generate an AP, we did not impose molecular constraints. When the magnitudes and activation/inactivation time constants of the voltage-gated conductances for Na^+^ and K^+^ are varied, either in pairs or simultaneously, we find that reductions in conductance have a limited ability to improve efficiency (Figure 3A,5), and reducing overlap by changing the kinetics of activation and inactivation is more effective (Figure 3B,5). Amongst these kinetic parameters, reducing the time constant for Na^+^ inactivation is the most effective (Figure 5).

We have extended the theoretical analysis of AP energy efficiency to six more models. This extension enabled us to compare the costs and efficiencies of different APs and examine how they depend on the properties of voltage-gated conductances. The final biophysical properties of ion channels produced by our optimization algorithm are within the biological bounds achieved in ion channels recorded experimentally: Even the fastest activation K^+^-channel time constant from our optimized models is only 50 µs (CA), which is within the limits of experimental data. For example, fast gating current time constants in voltage-gated ion channels of *Xenopus* oocytes have been reported from *Shaker* K^+^ channels (12 µs) [26], whilst mutant Na^+^ channels have been created with time constants of 0.07±0.02 ms [27].

The energy cost of an AP varies 22 fold, through differences in efficiency and height. Efficiency ranged from close to 100% in the mouse thalamocortical relay neuron model to 9% in the squid giant axon model at 6.3°C and was, as one might expect, determined by overlap (Figure 7). AP height determines the minimum capacitive Na^+^ load and this varied by a factor of 2.3 across models (Figure 2). Height may well be an important means of reducing energy consumption in nervous systems because the three lowest APs were also among the most efficient (Figure 1), but height has not received much attention as a variable cost factor.

Remarkably energy efficient APs, with total Na^+^ loads less than 1.3 times the minimum capacitive load, have recently been measured in 3 neurons, rat hippocampal mossy fibers [7] and mouse pyramidal cells in hippocampus (CA1) and neocortex [8]. Three of our seven models, RHI, RG and MTCR, achieved this same high level of efficiency (>70%), suggesting that energy efficient neurons are not uncommon in mammalian CNS. In agreement with the two earlier studies, these three models achieve a high efficiency by reducing the duration of the voltage-gated Na^+^ current to the point that there is little or no overlap with the voltage-gated K^+^ current, and this involves reducing the time constants for both Na^+^ activation and inactivation [7]. Operating at close to a rodent body temperature of 38°C must help to reduce time constants, but the mammalian models we ran were determined by experimental data gathered at room temperatures. Nonetheless, three of these models produced energy efficient APs. Our parameter variations (Figure 3C,D) confirm that overlap current is also reduced by reducing channel density within boundaries set by excitability, and Na^+^ inactivation is critical [7].

The importance of Na^+^ inactivation in reducing overlap current has also been demonstrated in mouse fast-spiking interneurons [8]. These cells use a fast and powerful voltage-activated K^+^ conductance to drive the falling phase of the AP down so quickly that there is very little Na^+^ inactivation, and this helps these fast-spikers to fire at high frequencies, but they pay a price in Na^+^ load. Because the Na^+^ conductance is not fully inactivated, there is a relatively long overlap. This type of fast-spiking AP is narrowed by advancing the repolarizing phase, leading to a longer overlap and lower efficiency. Consequently the shape of the AP waveform is a good predictor of efficiency – efficiency decreases as width narrows [8].

We find that the relationship between AP waveform and energy consumption is strongly model dependent (Figure 4). In the rat hippocampal interneuron model (RHI), AP energy consumption increases as width decreases (Figure 4B). In the squid giant axon model the relationship is reversed (Figure 4A) because the speed of Na^+^ inactivation is the major determinant of both AP width and overlap (Figure 5). Consequently reducing the inactivation time constant reduces the duration of both the AP and the overlap, leading to cost decreasing with decreasing width. This degree of model dependency shows that a trend that stands out among neurons whose APs are similarly configured for a specific purpose [8] can be reversed when APs are configured for a different purpose. Consequently, when one compares models of APs derived from a heterogeneous group of neurons, no trend is apparent (Figure 2).

Strong model dependency is a generic property of systems that use voltage-gated conductances to generate APs, and stems from the fact that the rate of change of membrane potential, and hence the behavior of voltage dependent conductances, depends on the sum of currents (the net current). This simple fact means that two large opposing voltage-gated conductances producing a massive Na^+^ overlap load can generate the same AP waveform as two smaller faster conductances with little overlap (Figure S1) – an example of many to one mapping. As expected from the many to one mapping of conductances onto voltage, and opposite relationships between waveform and energy, the sensitivity of AP efficiency to changes in conductance parameters is also strongly model dependent. Model dependency is apparent when one varies the same pairs of parameters in two models (Figure 3), but it was much more revealing to vary several parameters simultaneously. Why is this so?

The relationship between *N* conductance parameters and energy defines a surface in an *N*-dimensional space. When we vary a subset of parameters we get a limited impression of what is going on. For example, if we vary a single parameter we might cut across a valley in the landscape and see its low energy floor, but we do not see the floor dropping away sharply as one moves down the valley. Our search method explores the multidimensional energy landscape surrounding a particular model and (for it is easy to get lost in multi-dimensional space) it returns interpretable results. We learn how to adjust several parameters to move the model across the landscape from its starting point to the minimum energy value in its neighborhood, where the size of the neighborhood is defined by the boundaries of parameter variation. In comparison to systematic searches of parameter space (e.g. [28]), our directed search method is less computationally demanding.

The results of applying this search method demonstrate that it is advantageous to adjust several parameters simultaneously. All six models moved to their “neighborhood” minima by changing some of their core parameters, namely the total conductance, activation time constant and inactivation time constant of the voltage-gated Na^+^ conductance, and the total conductance and activation time constant of the voltage-gated K^+^ conductance. All models moved to their neighborhood minimum by adjusting their parameters to reduce the overlap between the Na^+^ and K^+^ currents (Figure 7). However, the signs and the relative magnitudes of the adjustments to the five parameters differed markedly, according to model (Figure 7A). For example, within the squid giant axon model, AP efficiency is improved by making relatively large changes to the Na^+^ activation and inactivation time constants, with lesser adjustments to the magnitudes of these conductances (Figure 5). By comparison, the cortical fast-spiking interneuron model reached its neighborhood minimum by reducing the magnitudes of both voltage-gated conductances and decreasing their time constants. These observations demonstrate that each model “sees” a different energy landscape in its neighborhood.

Adding conductances to a model changes the energy landscape. The crab motor neuron model contains an additional A-type K^+^ conductance (Figure S4). If the A-type conductance is fixed during optimization the minimized model's Na^+^ load is 11% higher than when all three conductances are free to vary within their bounds. Further energy savings should be possible through changes in other parameters affecting the Na^+^ and K^+^ currents. For example, the energy consumption can be decreased further by allowing the voltage dependence (slopes and mid-point voltages of Boltzmann functions) of the Na^+^ and K^+^ conductances to change (unpublished observation). This emphasizes that a large number of biophysical parameters contribute to the energy consumption of APs and that each of these may be tuned to produce greater energy efficiency, ultimately within boundaries set by the molecular dynamics of ions channels [25].

It is remarkable that, working within these boundaries, a number of energy efficient APs achieve efficiencies better than 70% [7], [8], [15] including 3 of our models (Figure 1). The energy consumption at rest can also have a considerable influence on energy usage and can, in some cases, be associated with the cost of generating signals [6], [24]. We found that, the energy consumption of the models at rest (calculated over the same period as the AP) was less than 1.5% of that consumed by the AP, irrespective of the AP's energy efficiency (Table S5). The advantage of high AP efficiency is clear [7]. The metabolic cost of APs has a significant influence on energy usage in neural tissue, sufficient to influence the function, design and evolution of nervous systems [1], [6]. A cubic millimeter of grey matter in cerebral cortex contains approximately 3 km of excitable axons, mainly formed by pyramidal neurons. Each pyramidal neurons uses approximately 4 cm of 0.3 µm diameter axon collateral to drive its 10,000 output synapses in grey matter and when its drives these synapses with an AP it incurs a minimum capacitive load of 2.36*10^8^ Na^+^ ions that requires approximately 8*10^7^ ATP molecules to eject [1]. Realizing that this minimum energy requirement is inflated by overlap, and noting that there was no data on the time course of currents generating APs in pyramidal neuron axon collaterals, Attwell and Laughlin (2001) adopted the squid overlap factor of 4 and calculated that AP transmission consumed significantly more energy than synaptic transmission. Energy efficient APs will reduce AP consumption by more than two thirds, so freeing up energy for processing [7]. How effective might they be?

**Figure 8:**
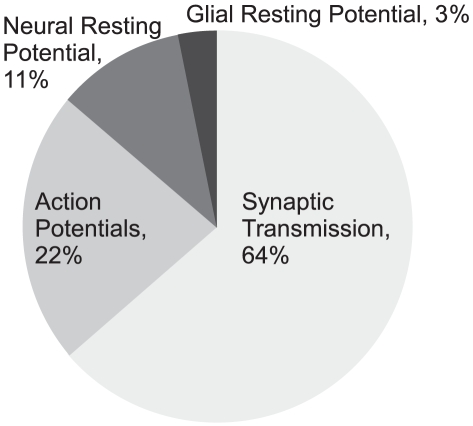
A revised energy budget for signaling in the grey matter of the rat brain. Incorporating the increased efficiency of action potentials in mammalian neurons into Attwell and Laughlin's [1] original energy budget for grey matter in the rat brain reduced the proportion of the energy budget consumed by action potentials. The proportion of the energy budget consumed by synaptic transmission is increased.

The effects of energy efficient APs on cortical processing can be gauged by recalculating Attwell and Laughlin's (2001) estimates, using the overlap factor of 1.2, found in mouse cortical pyramidal cells and assuming that the probability that a synaptic bouton releases a vesicle in response to an incoming spike remains at 0.25 [8] (Figure 1). Using neurons whose APs are 80% efficient, as opposed to the squid at 25%, has two pronounced effects. First, the level of signal traffic that can be supported by the specific metabolic rate of cortical grey matter increases by 60%, from an average firing rate of 4 Hz per neuron to 6.8 Hz. Second, higher AP efficiency shifts the balance of energy expenditure from APs to synapses (Figure 8). Thus improving the energy efficiency of APs enables nervous systems to devote more of their resources to information processing by synapses and permits higher rates of processing. Benefits of this magnitude could help to explain why a number of mammalian CNS neurons use energy efficient APs.

The apparent advantages of using energy efficient APs raises the obvious question, why do neurons produce APs with apparently wasteful overlap loads? A number of analyses of neuronal wiring patterns, circuit layouts and coding procedures demonstrate convincingly that nervous systems evolve to use space, materials and energy efficiently [6], [29]. Work on squid giant axon shows that this escape neuron puts overlap energy to good use, boosting conduction velocity [5]. Modeling demonstrated that an important determinant of overlap current, channel density, can be adjusted, together with axon diameter, to minimize the energy required to transmit an AP with a given velocity, and the native squid axon operates in this regime [4]. Despite the fact that this AP is used infrequently and overlap energy is invested in trying to survive “life or death” situations, the squid giant axon matches its components to use energy efficiently (c.f. [8]).

Given that energy is a limiting resource, it is highly likely that many other neurons produce APs with overlaps to improve the performance of their function. Carter and Bean [8] demonstrate this point by observing that the overlap current in their fast spikers is the price they pay for enabling high rates by avoiding Na^+^ inactivation. Comparative studies have revealed numerous examples of neurons in which conductance, and hence energy consumption, is increased to improve performance [6] and in fly photoreceptors the trade off between energy and performance has been measured and related to the basic biophysics of phototransduction [30]. In auditory and electrosensory pathways that are specialized to accurately code small time differences, axons with high channel density drive large synapses onto spherical neurons to reduce response time constants and noise [31]. These processes could well be matched to each other to distribute investment efficiently across the system, but ultimately energy is being invested in the contributions that these neurons make to behavior, with respect to the animal's fitness. It is possible therefore that in many neurons functional constraints and behavioral requirements prevent energy minimization – neurons pay for what behavior needs [6].

Given this overtly functional scenario, the task of looking more deeply into the efficiency of APs seems rather daunting. The variety of AP waveforms and conductances reflects the multitude of tasks they undertake [15], [32] and it is well established we must both measure and model APs to understand how they execute their tasks. Furthermore, these methods must account for the conductances that trigger APs and the conductances driven by APs, within the circuitry of the parent neuron. Is each neuron a special case or are their overarching principles? No matter what the answer to that question is, if we are to progress to an understanding of how conductances are organized to operate both effectively and efficiently we need to vary the parameters in these multi-compartment multi-conductance systems and observe the results. Our study of single compartment systems suggests that to reach this level of understanding the modeler, like the neuron, must be free to vary parameters simultaneously and obtain intelligible results in a reasonable time. Efficient optimization routines, perhaps built on those that we have trialed here, may be necessary for this endeavor.

## Methods

### Single compartment Hodgkin-Huxley model

We used single compartment deterministic Hodgkin-Huxley (HH) type models for our simulations. Since, seven different variants of Hodgkin-Huxley (HH) type models were used, we illustrate our methodology taking the squid axon model (SA) as an example.

The original Hodgkin-Huxley (SA) model contained two voltage-gated ion channels, Na^+^ and delayed rectifier K^+^ along with the leakage conductance. The dynamics of the membrane potential was governed by a set of activation and inactivation variables with the current balance equation having a form,
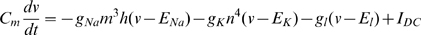
where, C_m_ is the membrane capacitance, g_Na_ is the conductance per unit area for the Na^+^ channel, g_K_ is the conductance per unit area for the K^+^ channel and g_l_ is the conductance per unit area for the leak channel. Parameter values used for the simulations are detailed in Table S1. I_DC_ is the constant current injection. The variables m, h and n follow first order kinetics of the type,
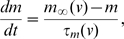
where, m_∞_(v) is the steady-state activation and τ_m_(v) is the voltage-dependent time constant. To allow for the change of shape of APs we introduce scaling terms for the time constants of the channels without altering the voltage dependence of the channels i.e., the activation and inactivation functions now read:
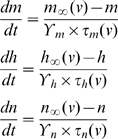
where, 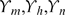 are the speed factors (scaling terms) for the channels, that determine the speed of the underlying kinetics. Similarly, conductance per unit area for the Na^+^ and K^+^ channels have scaling factors that pre-multiply them to either increase or decrease the conductance per unit area. The crab leg axon (CA) neuron was modeled as in [9]. Similar approach was adhered for the mouse fast-spiking (MFS) cell model with parameters taken from [10]. The bee Kenyon cell (BK) was modeled after [16]. The rat hippocampal interneuron (RHI) model had parameters from [14]. Similarly, the rat granule cell (RG) was modeled after [13] and the mouse thalamo-cortical relay neuron (MTCR) was modeled after [11]. All parameter values are detailed in Table S1.

Temperature was factored into the single compartment models using a normalization term (ϕ) for the time constants of the respective channels:
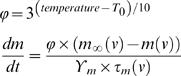
where T_0_ is the temperature at which the experiments were performed.

### Optimization

Many neurons are excitable; quiescent at rest, they exhibit strong, sustained oscillations if driven—these oscillations are the APs of the neuron. Current driving the neuron typically causes a transition (dynamical bifurcation) from the resting state to a periodically spiking state. However, not all combinations of biophysical parameters, such as the peak conductances, time constants, or the voltage dependencies will give rise to periodic spiking. As parameter shifts can cause sudden transitions in the dynamics, extra care is needed in any optimization procedure. We use standard linear analysis to test for the presence of a periodic orbit in the single compartment Hodgkin-Huxley type models [33]. Once a periodic orbit is obtained, numerical continuation techniques [34] can be used to follow the change in the orbit and its period as parameters are made to vary; in general, to allow for large parameter shifts, we numerically solve the underlying differential equations for each new parameter set.

In our case, the optimization objective is to minimize the integral of the inward (Na^+^) current over the oscillation period, i.e., 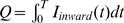, subject to a fixed amplitude of the oscillation in the membrane potential. Here, 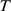 is the oscillation period, which itself depends on the parameters. 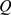 is undefined for parameter sets for which no oscillations result because 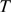 is not well-defined; such an occurrence must be handled separately during optimization.

Optimization is performed via a gradient-free, simplex-based direct search method known as the Nelder-Mead algorithm. For optimizing a function in two variables, the simplex is a triangle. The algorithm compares the function values at the three vertices of the triangle and reflects the triangle away from the worst vertex, shrinks it towards the best vertex, or expands or contracts the triangle [35]. The soft constraint on the AP height is implemented as a quadratic loss function added to the ionic load [36], [37]. Any parameter set that does not lead to oscillations immediately becomes the worst vertex. Alternatively, we search for the global minimum of the ionic load by using a multi-start, hill-climbing algorithm based on Newton's method [37] that is designed to be robust to local minima [38]. Both algorithms converged to the nearly the same set of parameter values. In contrast, random search, simulated annealing and algorithms based on differential evolution yielded sub-optimal results.

We optimized the neuron models by scaling the peak conductances and time constants, either jointly or separately, subject to the constraints in Tables S3. In particular, we scaled the time constants for fast sodium activation, inactivation, and the delayed rectifier potassium activation variables. In those cases for which sodium activation is assumed to be infinitely fast (MFS and RHI models), only the two remaining time constants were scaled.

Custom code was written in Wolfram Mathematica 7.0 (Wolfram Research Inc, USA) and Matlab 2009a (The MathWorks Inc, USA) for performing and analyzing the simulations and optimization. Standard optimization routines available in the Optimization toolbox in Matlab 2009a were used to cross-check results obtained in Mathematica. To study the dynamics both quantitatively and qualitatively, numerical continuation code was implemented using AUTO [34], MATCONT [39] or custom code in Wolfram Mathematica 7.0 (Wolfram Research Inc, USA).

### Calculation of energy and efficiency

Energy consumption in our model is defined as the amount of Na^+^ ions consumed during an AP. The Na^+^/K^+^ pump hydrolyses one ATP molecule for every three Na^+^ ions exported and two K^+^ ions imported from/to the cell [3]. We compute this charge in response to a constant current injection. This is done by integrating the area under the Na^+^ current curve (for a single period), which defines the Na^+^ load of a single AP. Number of ATP molecules used can be calculated by multiplying this number by N_A_/(3.F), where N_A_ is the Avagadro's constant and F is the Faraday's constant.

### Gating capacitance model

The original Hodgkin-Huxley model ignores the transient change in capacitance caused due to charge displacement during the activation (outward) and the inactivation (inward) of the channel. The gating current becomes prominent during the rising foot of the AP. In modeling this effect of transient capacitance, we follow Crotty and Levy [23] and Sangrey *et al.* [40] and describe the gating current as a voltage dependent capacitive current. The total membrane capacitance is the sum of this voltage dependent capacitance and the original voltage independent membrane capacitance, C_0_ (0.88 µF cm^−2^) [41]. The voltage dependent capacitance has a maximum value of 1 nF mS^−1^. In our analysis, we have ignored the contribution of the gating charge due to inactivation of the Na^+^ channel and the K^+^ channel. The membrane voltage of the compartment is determined via the following equation,
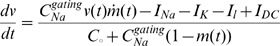



## Supporting Information

Figure S1 Single action potentials with same shape can be produced by different Na^+^ and K^+^ currents incurring different energy costs. (A) The waveforms of two action potentials (red and black) with similar height and width. (B) The Na^+^ (black, solid) and K^+^ (black, dashed) currents producing action potential 1 (black) waveform in A. The action potential has a Na^+^ load of 2244 nC cm^−2^. (C) The Na^+^ (red, solid) and K^+^ (red, dashed) currents producing action potential 2 (red) waveform in A. The action potential has a Na^+^ load of 1275 nC cm^−2^.
(0.23 MB EPS)

Figure S2 Shorter action potentials consume less energy. (A) Two action potentials from the squid giant axon model after optimization constrained to the original height (grey, solid) and constrained to a lower height than the original (black, dashed). (B) The cumulative Na^+^ load of the action potential constrained to the original height (grey, solid) and constrained to a lower height than the original (black, dashed). (C) The Na^+^ (grey, dashed) and K^+^ (dark grey, solid) currents producing the shorter action potential. (D) The amount of energy consumed by a single action potential after optimization with four different height constraints.
(0.22 MB EPS)

Figure S3 The effect of gating charge on the optimization of the squid giant axon model. (A) Two action potential waveforms after optimization without (black) or with (grey) the gating capacitance. (B) The cumulative Na^+^ load during each action potential without (black) or with (grey) the gating capacitance. (C) The underlying Na^+^ (grey, dashed) and K^+^ (grey, solid) currents producing the optimized action potential with gating capacitance.
(0.21 MB EPS)

Figure S4 The effect of additional conductances on action potential energy consumption after optimization. (A) Three action potentials from the crab motor neuron (CA) model. The original action potential (red), the action potential after optimization when parameters of the A-type K^+^ voltage-gated channels were fixed (black) and the action potential after optimization when parameters of the A-type K^+^ voltage-gated channels were also allowed to vary (blue). (B) The three currents from the original crab motor neuron (CA) model. (C) The three currents from the optimized crab motor neuron (CA) model when parameters of A-type currents were fixed. (D) The three currents from the optimized crab motor neuron (CA) model when parameters of A-type currents were allowed to vary.
(0.33 MB EPS)

Figure S5 The overlap between Na^+^ and K^+^ currents are reduced after optimization. (A) The Na^+^ (black, dashed) and K^+^ current (black, solid) of the crab motor neuron (CA) model before optimization. (B) The Na^+^ (grey, dashed) and K^+^ current (grey, solid) of the crab motor neuron (CA) model after optimization. (C) The Na^+^ (black, dashed) and K^+^ current (black, solid) of the mouse fast spiking neuron (MFS) model before optimization. (B) The Na^+^ (grey, dashed) and K^+^ current (grey, solid) of the mouse fast spiking neuron (MFS) model after optimization.
(0.21 MB EPS)

Table S1 Parameters of action potentials from the seven single compartment models.
(0.05 MB DOC)

Table S2 Properties of action potential waveform of single action potentials from the seven single compartment models, after introduction of ±5% error on peak conductance values of Na^+^, K^+^ (delayed-rectifier) and leak conductances.
(0.03 MB DOC)

Table S3 Optimal parameter set when all parameters of the Na^+^ and K^+^ channels are allowed to vary.
(0.04 MB DOC)

Table S4 Comparison between original squid axon model (SA) and the modified squid axon model (HHSFL) at 6.3°C.
(0.03 MB DOC)

Table S5 Resting and signaling costs from the seven single compartment models.
(0.03 MB DOC)
