# Supplementary material for: Action Potential Energy Efficiency Varies Among Neuron Types in Vertebrates and Invertebrates
Source: PLoS Comput Biol. 2010 Jul 1;6(7):e1000840. doi: 10.1371/journal.pcbi.1000840 (PMC2895638; doi:10.1371/journal.pcbi.1000840)
Supplement: Table S1 — Parameters of action potentials from the seven single compartment models. (0.05 MB DOC) [file pcbi.1000840.s006.doc]

|  | **SA** | **CA** | **MFS** | **BK** | **RHI** | **RG** | **MTCR** |
| --- | --- | --- | --- | --- | --- | --- | --- |
| **Area [cm^2^]** | 1 | 1 | 1 | 1 | 1 | 1 | 1 |
| **Specific membrane capacitance [μF cm^-2^]** | 1 | 1 | 1 | 1 | 1 | 1 | 1 |
| **Leak reversal potential [mV]** | -54.4 | -17 | -70 | -81 | -65 | -70 | -70 |
| **Na^+^ reversal potential [mV]** | 50 | 55 | 50 | 58 | 55 | 55 | 50 |
| **K^+^ reversal potential [mV]** | -77 | -72 | -90 | -81 | -90 | -90 | -90 |
| **K^+^ reversal potential (a-type) [mV]** | - | -75 | - | - | - | - | - |
| **Ca^2+^ reversal potential [mV]** | - | - | - | - | - | 80 | 0 |
| **Leak conductance [mS cm^-2^]** | 0.3 | 0.3 | 0.25 | 0.075 | 0.1 | 0.03 | 0.05 |
| **Na^+^ conductance (I) [mS cm^-2^]** | 120 | 120 | 112.5 | 35 | 35 | 55 | 3 |
| **Na^+^ conductance (II) [mS cm^-2^]** | - | - | - | 3 | - | - | - |
| **H conductance [mS cm^-2^]** | - | - | - | - | - | 0.03 | - |
| **K^+^ conductance [mS cm^-2^]** | 36 | 20 | 225 | 1.5 | 9 | 9 | 5 |
| **K^+^ conductance (a-type) [mS cm^-2^]** | - | 47.7 | - | 14.5 | - | 1.2 | - |
| **K^+^ conductance (slow transient) [mS cm^-2^]** | - | - | 0.39 | 2 | - | - | - |
| **K^+^ conductance (c-type) [mS cm^-2^]** | - | - | - | - | - | 18 | - |
| **Ca^2+^ conductance (l-type) [mS cm^-2^]** | - | - | - | - | - | 0.9 | - |
| **Ca^2+^ conductance (t-type) [mS cm^-2^]** | - | - | - | - | - | - | 5 |
