# Supplementary material for: Action Potential Energy Efficiency Varies Among Neuron Types in Vertebrates and Invertebrates
Source: PLoS Comput Biol. 2010 Jul 1;6(7):e1000840. doi: 10.1371/journal.pcbi.1000840 (PMC2895638; doi:10.1371/journal.pcbi.1000840)
Supplement: Table S2 — Properties of action potential waveform of single action potentials from the seven single compartment models, after introduction of ±5% error on peak conductance values of Na+, K+ (delayed-rectifier) and leak conductances. (0.03 MB DOC) [file pcbi.1000840.s007.doc]

|  | **SA** | **CA** | **MFS** | **BK** | **RHI** | **RG** | **MTCR** |
| --- | --- | --- | --- | --- | --- | --- | --- |
| **Overestimation of Na^+^ load [nC cm^-2^]** | 1161.7 | 374 | 329.4 | 231.2 | 127.7 | 76.6 | 65.5 |
| **Underestimation of Na^+^ load [nC cm^-2^]** | 1034.5 | 353.6 | 298 | NA | 119.3 | 75.5 | 61.1 |
| **Overestimation of AP height [mV]** | 99.7 | 108.5 | 129.8 | 61.6 | 99.3 | 70 | 66.8 |
| **Underestimation of AP height [mV]** | 97.7 | 102.1 | 129.8 | NA | 93.9 | 66.7 | 63.6 |
| **Overestimation of AP width [ms]** | 1.5 | 0.9 | 0.5 | 2.5 | 0.6 | 0.2 | 1.1 |
| **Underestimation of AP width [ms]** | 1.5 | 0.9 | 0.5 | NA | 0.6 | 0.2 | 1.1 |
