# Supplementary material for: Action Potential Energy Efficiency Varies Among Neuron Types in Vertebrates and Invertebrates
Source: PLoS Comput Biol. 2010 Jul 1;6(7):e1000840. doi: 10.1371/journal.pcbi.1000840 (PMC2895638; doi:10.1371/journal.pcbi.1000840)
Supplement: Table S3 — Optimal parameter set when all parameters of the Na+ and K+ channels are allowed to vary. (0.04 MB DOC) [file pcbi.1000840.s008.doc]

|  | **Minimum**  **bound** | **Maximum**  **bound** | **SA** | **CA** | **MFS** | **BK** | **RHI** | **MTCR** |
| --- | --- | --- | --- | --- | --- | --- | --- | --- |
| **Height constraint [mV]** |  |  | 98.7 | 106.5 | 129.3 | 61 | 55.5 | 65 |
| **Direct current injection [μA cm^-2^]** |  |  | 20 | 20 | 20 | 4 | 20 | 2 |
| **Na^+^ conductance scaling (I)** | 0.3 | 4 | 0.91 | 0.54 | 2 | 1.14 | 0.87 | 0.9 |
| **Na^+^ conductance scaling (II)** | 0.3 | 4 | - | - | - | 0.46 | - | - |
| **K^+^ conductance scaling** | 0.3 | 4 | 0.3 | 1.93 | 0.3 | 0.62 | 2.9 | 1.12 |
| **Na^+^ activation scaling (I)** | 0.3 | 2.5 | 0.3 | 0.3 | - | 0.3 | - | - |
| **Na^+^ inactivation scaling (I)** | 0.3 | 2.5 | 0.3 | 0.56 | 0.3 | 1.5 | 0.51 | 1.1 |
| **Na^+^ activation scaling (II)** | 0.3 | 2.5 | - | - | - | 1.11 | - | - |
| **Na^+^ inactivation scaling (II)** | 0.3 | 2.5 | - | - | - | 0.92 | - | - |
| **K^+^ activation scaling** | 0.3 | 2.5 | 0.88 | 2.47 | 0.3 | 0.52 | 0.88 | - |
