# Supplementary material for: Action Potential Energy Efficiency Varies Among Neuron Types in Vertebrates and Invertebrates
Source: PLoS Comput Biol. 2010 Jul 1;6(7):e1000840. doi: 10.1371/journal.pcbi.1000840 (PMC2895638; doi:10.1371/journal.pcbi.1000840)
Supplement: Table S4 — Comparison between original squid axon model (SA) and the modified squid axon model (HHSFL) at 6.3°C. (0.03 MB DOC) [file pcbi.1000840.s009.doc]

|  | **Squid axon model (SA)** | **Modified Squid axon model (HHSFL)** |
| --- | --- | --- |
| **Original Na^+^ load [nC cm^-2^]** | 1098 | 681.6 |
| **Original AP height [mV]** | 98 | 100.8 |
| **Original AP full-width at half maximum [ms]** | 1.47 | 1.2 |
| **Optimum Na^+^ load [nC cm^-2^]** | 263 | 109 |
| **Optimum AP full-width at half maximum [ms]** | 0.8 | 0.7 |
| **Capacitive minimum [nC cm^-2^]** | 98 | 100.8 |
