# Supplementary material for: Action Potential Energy Efficiency Varies Among Neuron Types in Vertebrates and Invertebrates
Source: PLoS Comput Biol. 2010 Jul 1;6(7):e1000840. doi: 10.1371/journal.pcbi.1000840 (PMC2895638; doi:10.1371/journal.pcbi.1000840)
Supplement: Table S5 — Resting and signaling costs from the seven single compartment models. (0.03 MB DOC) [file pcbi.1000840.s010.doc]

|  | **SA** | **CA** | **MFS** | **BK** | **RHI** | **RG** | **MTCR** |
| --- | --- | --- | --- | --- | --- | --- | --- |
| **Resting^†^ Na^+^ Load [nC cm^-2^]** | 14.15 | 0.12 | * | 0.0001 | 1.09 | 0.09 | 0.04 |
| **AP Na^+^ Load [nC cm^-2^]** | 1098 | 364 | 315 | 186 | 76 | 72 | 65 |

^†^ The resting costs were calculated over the same period as the action potential giving a direct comparison without any assumption about the spike rates of the neurons.

* Due to regular spiking in the MFS cell at rest, the energy consumption in the absence of APs cannot be calculated.
